# Gender and the impact of COVID-19 on demand for and access to health care: Intersectional analysis of before-and-after data from Kenya, Nigeria, and South Africa

**DOI:** 10.7189/jogh.12.05024

**Published:** 2022-08-13

**Authors:** Safa Abdalla, Elizabeth G Katz, Angela Hartley, Gary L Darmstadt

**Affiliations:** Global Center for Gender Equality, Department of Pediatrics, Stanford University School of Medicine, Stanford, California, USA

## Abstract

**Background:**

Global health emergencies can impact men and women differently due to gender norms related to health care and social and economic disruptions. We investigated the intersectionality of gender differences of the impact of COVID-19 on health care access with educational and socio-economic factors in Kenya, Nigeria, and South Africa.

**Methods:**

Data were collected by Opinion Research Business International using census data as the sampling frame. We used conditional logistic regression to estimate the change in access to health care after the emergence of the pandemic among men and women, stratified by educational level. We also examined the change in demand for various health care services, stratified by self-reported experiences of financial difficulty due to the pandemic.

**Results:**

Among those reporting a need to seek health care in South Africa, there was a statistically significant decline in the ability to see a health care provider during the pandemic among women, but not among men; this gender gap was more evident in those who did not have post-secondary education (odds ratio (OR) = 0.08, *P* = 0.041 among women; no change among men) than for those with post-secondary education (OR = 0.20, *P* = 0.142 among women; OR = 0.50, *P* = 0.571 among men). South African women financially affected by the pandemic had a significant decline in seeking preventive care during the pandemic (OR = 0.23, *P* = 0.022). No conclusive effects were noted in Nigeria or Kenya.

**Conclusions:**

In South Africa, the pandemic and its strict control measures have adversely and disproportionately impacted disadvantaged women, which has implications for the nature of the long-term impact as well as mitigation and preparedness plans.

Global health emergencies can have disparate impacts by gender due to prevailing norms or the resulting economic and social upheaval [1]. The disruptions caused by the current pandemic and the strict measures implemented to control it have become clearer since its emergence in early 2020, spanning multiple domains, sometimes with clear gender differentials. According to a study by the World Bank, more women than men lost income because they had to stop working [2]. Widespread disruptions to health care availability and demand have also been a hallmark, and by virtue of the unique health care needs of women during pregnancy and childbirth, are thought to have had a higher impact among women than men [3].

The impact of the pandemic has been felt in all parts of the world, in high-, middle- and low-income countries, and despite more than 2 years since its onset and ongoing mitigation and vaccination efforts, emerging variants and surging cases continue to force authorities in many countries to maintain or re-establish stringent control measures. However, it remains unclear whether policies aimed to address the indirect impacts on health services have been appropriately targeted to those most in need of them, particularly in places where health systems are already significantly challenged [3]. This is partly due to limited in-depth interrogation of potential impacts and the responsible pathways. Data are therefore central to inform response, recovery, and future preparedness. This is crucial for gender equality, as shocks caused by the pandemic can prompt efforts that address historically structural barriers to gender equality but may also uncover and exacerbate existing inequalities if they enable the expression of restrictive norms and sanctions. Thus, it is important to examine, potentially anticipate, and promote positive change while mitigating negative impacts.

While several studies have identified declines in various health care services due to the pandemic and its control measures [4-11], relatively few studies have explicitly examined their impact on genders. Some of the gendered impacts are represented in the exclusivity of some of the disrupted services to women, such as maternal health services, family planning, and pregnancy termination [12-14]. Gendered disruptions to health care access were documented in multiple non-peer-reviewed reports showing a more pronounced decline in access to health care among women than men after the onset of the COVID-19 pandemic [15,16]. This pattern was evident in sub-Saharan Africa and in Latin America and the Caribbean (Figure S1 in the **Online Supplementary Document**). Country-level analysis of national data from Kenya and South Africa revealed a similar pattern [16]. A smaller-scale study in Kenya noted that women were more likely to forego health care [17], while another study in Kwa-Zulu Natal in South Africa found no change in utilisation of ambulatory care among men or women [18]. This indicates that national-level patterns may belie patterns in specific groups or geographical areas. We aimed to investigate the intersection of gender differentials in the impact of the pandemic on health care access with educational and socio-economic factors using data from Kenya, Nigeria, and South Africa. We sought to understand the limitations of available data when stress-tested with intersectional gender analysis, examining whether gender differentials in the decline in health care access were concentrated in a specific socio-economic group, and whether the financial impacts of the pandemic affected the demand for health care services.

## METHODS

### Data

We used data from the COVID-19 Health Services Disruption Survey 2020, designed by the Institute for Health Metrics and Evaluation (IHME) in collaboration with the Bill and Melinda Gates Foundation (BMGF), described in detail elsewhere [19,20]. Briefly, the data were collected by Opinion Research Business International (ORB) using computer-associated telephone interviews (CATI) in Kenya, Nigeria, and South Africa from July 6, 2020, to July 23, 2020. ORB used regional, gender, and age quotas that corresponded with the most recent national census to draw a randomly selected, nationally representative sample in each country. The sample included only those who had a phone. Surveyors conducted 3058 interviews in total (1002 in Kenya, 1016 in Nigeria, and 1040 in South Africa). All collected data went through an extensive quality control process consisting of daily data downloads, audio checks, interview duration checks, and compliance with sampling requirements. The overall response rate was 87% in South Africa, 72% in Nigeria, and 68% in Kenya. Weights were calculated to correct for a slight age imbalance among respondents from Kenya and for slight age and gender imbalances among respondents from Nigeria, while the achieved sample in South Africa aligned with Census data and did not require weights.

Participants reported their retrospective experiences with demand for and access to health care from December 2019 to February 2020, prior to the pandemic surge (baseline), and from March to July 2020, in the midst of the pandemic. Specifically, the participants were asked: “Were you able to see a healthcare provider during December – February/ since March?” Response options were: Yes, I saw a provider each time I needed to; Partly – I saw a provider during this time, but not every time I needed; or No – I did not see a provider. Respondents were also asked about the conditions they sought care for or the specific services they sought and the reasons for not accessing health care when they felt they needed it [21]. We were unable to analyse the latter at the level of granularity we were seeking due to sample size limitations.

### Analysis

#### Sociodemographic background

We used the χ^2^ test and the *t* test for independent samples to compare the sociodemographic characteristics of men and women in each country, and to analyse the association of experience of financial hardship due to the pandemic and its control measures with educational level and initial income before the onset of the pandemic. Baseline income was categorised into terciles, producing 5000 and 16 000 cut-off values in Kenya, 10 000 and 33 000 cut-off values in Nigeria, and 3000 and 7300 cut-off values in South Africa, in the local currency of each country.

#### Access to health care

This analysis included only those reporting a need to see a health care provider both before and after the onset of the pandemic. We grouped the responses to whether the participants were able to see a health care provider into two categories: “Yes” if they were able to see a provider every time they needed to, and “No” if they were not able to see one at any time they needed to. We used conditional logistic regression, with the respondent as their own control, to examine the change in demand for access to health care between the time before and after the onset of the pandemic. Survey weights were applied where applicable. The analysis was stratified by the respondents’ education level grouped into two categories: none, primary, or secondary (12 or fewer years of education) and post-secondary (more than 12 years of education).

#### Demand for health care

This analysis included all participants who had a valid response to the question on whether they needed to see a health care provider, asked regarding the period before and after the onset of the pandemic. We grouped some of the conditions for which health care was sought: heart disease, stroke, cancer, diabetes, kidney disease, asthma, liver disease, alcohol, high cholesterol, high blood pressure, hearing, vision, bone-joint conditions, mental health, and injury were classified as non-communicable diseases; tuberculosis, HIV, malaria, and pneumonia were classified as non-COVID communicable diseases. Preventive health care and sexual health care were kept as separate categories. COVID-19 was excluded due to its clear time dependence; the distinction of other lung diseases and minor health conditions as either communicable or non-communicable could not be ascertained. We classified demand for each group into a yes-no variable, where “no” included everyone who did not express a need for care for the specific group of conditions, including those not expressing a need for health care for any reason. We used conditional logistic regression to examine the change in demand for preventive care, care for non-communicable conditions, and sexual health care. The analysis was re-run in the group reporting financial difficulty due to the COVID-19 pandemic and/or efforts to control it. Sample sizes for those who were not financially impacted were too small for models to run successfully. All analyses excluded cases with missing values for the variables included in each analysis.

The analysis was carried out with SAS 9.4 and an alpha cut-off of 0.05 was used to determine statistical significance.

## RESULTS

In Kenya, women and men were similar in age, educational level, and experience of financial hardship due to the pandemic, but women were more likely than men to be in the lowest income tercile (38% vs 25%, *P* < 0.001) and to be single, widowed, or divorced (49% vs 45%, *P* = 0.043). A similar pattern was evident in Nigeria and South Africa, except that in South Africa, women also had higher mean age and a higher proportion had less than a tertiary-level education than men (**Table 1**). Financial hardship due to the pandemic was more common among women with lower educational attainment in Kenya and Nigeria (Table S1 in the **Online Supplementary Document**). Financial hardship also varied by initial income in Kenya and among both women and men in South Africa.

**Table 1:** Background characteristics of respondents in Kenya, Nigeria, and South Africa, 2020

Characteristics	Men	Women	*P*-value*
**Kenya (Men n = 494, Women n = 508)**			
Age in years: mean (SD)	36 (1.0)	35 (0.8)	0.389
None, primary, or secondary education	42%	45%	0.397
% in the lowest income tertile	26%	38%	<0.001
Financial hardship due to pandemic	89%	88%	0.742
Single (single, widowed, divorced)†	45%	49%	0.043
**Nigeria (Men n = 550, Women weighted n = 466)**			
Age in years: mean (SD)	36(0.8)	34(0.9)	0.249
None, primary, or secondary education	39%	43%	0.226
% in the lowest income tercile	22%	42%	<0.001
Financial hardship due to pandemic	91%	89%	0.318
Single (single, widowed, divorced)	57%	50%	0.048
**South Africa (Men n = 475, Women weighted n = 565)**			
Age in years: mean (SD)	38(0.5)	39(0.5)	0.020
None, primary, or secondary education	60%	66%	0.038
% in the lowest income tercile	24%	44%	<0.001
Financial hardship due to pandemic†	70%	67%	0.321
Single (single, widowed, divorced)‡	50%	59%	0.004

SD – standard deviation

*χ2 test for categorical variables, *t* test for numeric variables.

†Missing = 1 woman.

‡Missing = 1 man and 1 woman.

**Figure 1:**
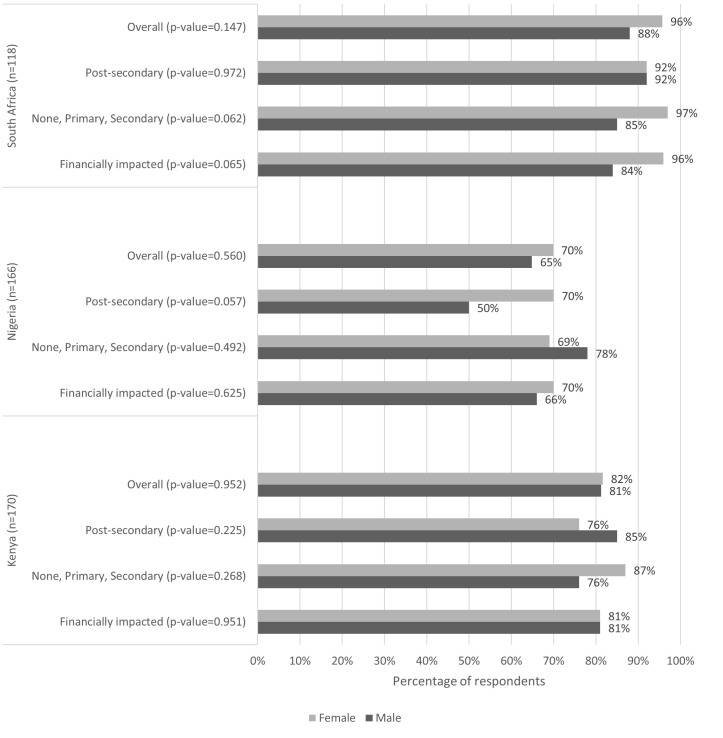
Baseline access to health care from December 2019 to February 2020 in Kenya, Nigeria, and South Africa.

Prior to the onset of the pandemic, reported health care access was largely high and similar among women and men with some differences in education in South Africa and Nigeria on the margin of statistical significance (**Figure 1**). There was a statistically significant decline in access to health care among women overall (odds ratio (OR) = 0.12, *P* = 0.004) and among women with lower education (none, primary, or secondary) (OR = 0.08, *P* = 0.017) in South Africa (**Table 2**). In Kenya and Nigeria, the change in health care access was not statistically significant, despite prominent patterns depicting a decline in access to health care for men and women in Kenya and an increase in access among both men and women with post-secondary education in Nigeria.

**Table 2:** Change in access to health care between the period from December 2019 to February 2020 and the period from March 2020 to July 2020 in Kenya, Nigeria, and South Africa

Country	Gender	Education	Odds ratio	*P*-value
Kenya	Men (n = 8)	None, Primary, Secondary	1.00	1.000
	Women*	None, Primary, Secondary	0.09	0.178
	Men (n = 17)	Post-secondary	0.35	0.147
	Women (n = 9)	Post-secondary	0.58	0.447
	Men	Overall	0.41	0.169
	Women	Overall	0.28	0.067
Nigeria	Men	None, Primary, Secondary	1.01	0.987
	Women (n = 6)	None, Primary, Secondary	0.79	0.825
	Men (n = 8)	Post-secondary	4.10	0.125
	Women (n = 14)	Post-secondary	3.31	0.139
	Men	Overall	1.76	0.225
	Women	Overall	1.82	0.287
South Africa	Men*	None, Primary, Secondary	1.00	1.000
	Women (n = 13)	None, Primary, Secondary	0.08	0.017
	Men*	Post-secondary	0.50	0.571
	Women (n = 6)	Post-secondary	0.20	0.142
	Men	Overall	0.67	0.657
	Women	Overall	0.12	0.004

n – number with change in either direction

*n <5.

**Figure 2:**
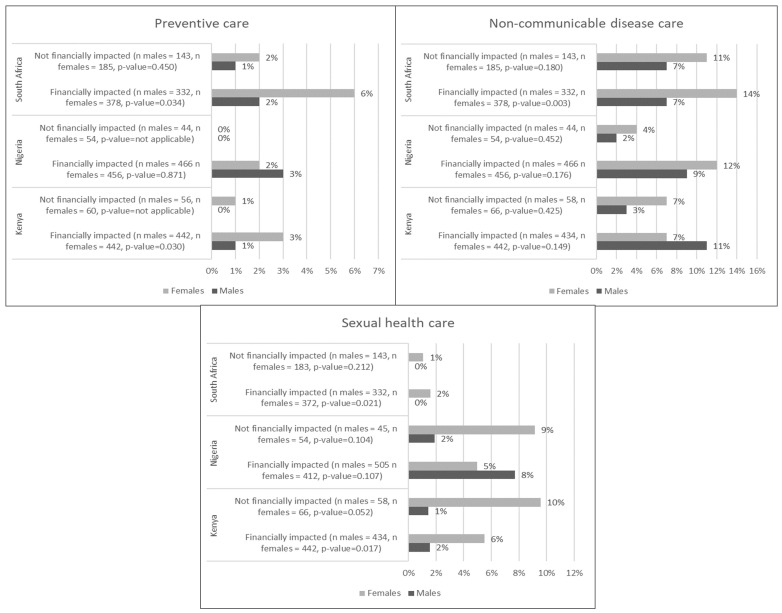
Baseline demand for preventive, non-communicable disease, and sexual health care (December 2019 to February 2020) in Kenya, Nigeria, and South Africa.

A small percentage of respondents indicated demanding preventive care before the pandemic’s onset, with more women than men indicating so in South Africa and Kenya, most notably among those who ended up having pandemic-related financial hardship (**Figure 2**). In South Africa, there was a statistically significant decline in demand for preventive care among women with financial hardship (OR = 0.23, *P*-value = 0.022), but not among men (**Table 3**). Baseline demand for care for non-communicable conditions was similar for men and women, except in South Africa, where it was significantly higher among women than men with financial hardship (14% vs 7%, *P* = 0.003) (**Figure 2**). However, there was no evidence of change in demand for care for non-communicable conditions in any group in the three countries during the pandemic (**Table 3**). Baseline demand for sexual health care was particularly low in South Africa (2% or less) and was significantly higher for women than men in Kenya (**Figure 2**) but was unchanged from baseline during the pandemic (**Table 3**).

**Table 3:** Change in demand for preventive care, care for non-communicable diseases, and sexual health care between the period from December 2019 to February 2020 and the period from March 2020 to July 2020 overall and among the financially impacted in Kenya, Nigeria, and South Africa*

Country	Gender	OR overall	*P*-value	OR – financially impacted	*P*-value
**Preventive care**					
Kenya	Men	1.00	1.00	1.00	1.000
	Women	2.07	0.141	2.23	0.132
Nigeria	Men	1.40	0.475	1.40	0.475
	Women	0.75	0.663	0.75	0.663
South Africa	Men	0.86	0.782	0.67	0.530
	Women	0.47	0.096	0.23	0.022
**Non-communicable diseases**					
Kenya	Men	1.11	0.773	1.20	0.631
	Women	1.08	0.851	1.12	0.801
Nigeria	Men	0.85	0.627	0.92	0.796
	Women	0.65	0.270	0.67	0.313
South Africa	Men	0.61	0.143	0.67	0.321
	Women	0.67	0.183	0.88	0.715
**Sexual health care**					
Kenya	Men	NA	NA	NA	NA
	Women	0.72	0.290	0.84	0.569
Nigeria	Men	0.70	0.282	0.66	0.230
	Women	0.66	0.239	0.66	0.273
South Africa	Men	NA	NA	NA	NA
	Women	1.20	0.763	1.00	1.000

NA – at least one parameter for calculating odds ratio = 0, OR – odds ratio

*Odds ratio (OR) of more than 1 means an increase in demand for care from March 2020 to July 2020 compared with the period from December 2019 to February 2020. OR less than one means a decrease in demand for care from March 2020 to July 2020 compared with December 2019 to February 2020.

## DISCUSSION

The study revealed signals of the gendered impact of the pandemic on demand and access to health care in South Africa with a clear intersection between socio-economic disadvantage and gender. There was no conclusive evidence of similar intersections in Kenya or Nigeria.

We were unable to explore the reported reasons for the pandemic’s differential impact on health care demand and access in South Africa, though the country’s implementation of one of the strictest national lockdowns in the world likely played a role [22,23]. Despite health facilities remaining open, their initial lockdown imposed highly stringent movement restrictions, including a curfew from the evening to early morning and restrictions on public transport times and capacity as well as hikes in costs [9]. As education can proxy income level, restrictions on public transport meant that those with financial means could afford private transport and better health care access. In addition, South Africa lessened restrictions around the practice of telemedicine to improve access to health care and mHealth initiatives via SMS, and smartphone apps were deployed in the management of communicable and non-communicable diseases [24,25]. Internet access and smartphone ownership are known to disadvantage women in South Africa [26], and women with limited education may have been particularly excluded [27,28]. Another reason could be differentials in access to information about the extent of restrictions (or lack thereof) on health facilities. Public preventive services in South Africa are free [29] and therefore inability to pay may not fully explain why they were deprioritized by women financially impacted by the pandemic. Medical insurance afforded by the wealthy, however, may have offered a safety net for faster and more accessible care [9]. It is also possible that physical access due to restrictions on public transport or hikes in transport costs led women to forego services that they thought could be postponed [9]. While all these factors would affect men as well, the larger impact on women may have been the result of higher poverty levels among women and/or norms restricting their financial decision-making. Fear of contracting COVID-19 was frequently cited as a reason for the decline in demand for health care [9,30], although it is not clear whether it was responsible for lower health care access among economically disadvantaged women.

There were some differences in the pandemic response of Kenya and Nigeria compared with South Africa that may explain the more conclusive differentials in the latter. In contrast to the national strict lockdown in South Africa, Nigeria implemented a lockdown gradually in late March 2020, starting with three of the 36 states, and expanding subsequently to the rest [31,32]. In addition, even when the easing of the initial lockdown happened concurrently in both countries, South Africa’s lockdown remained stricter than Nigeria’s [33]. Similar to Nigeria, the lockdown in Kenya was gradual and involved only virus hotspots initially. It is, therefore, possible that the stricter lockdown in South Africa led to larger and more conclusive effects than in Kenya and Nigeria. The apparent increase in health care access among men and women with post-secondary education in Nigeria, albeit not statistically significant, is unexpected. If true, it could indicate possible precautionary overuse of health services during the pandemic by those who could afford them. However, given the small sample sizes for this analysis, results should be viewed as hypothesis-generating.

As the pandemic continues to constantly challenge health systems, our findings bring to the forefront multiple intersecting axes of social determinants of health and context-specific nuances in the discourse on universal health care. The United Nations’ call for universal health care coverage at the time of COVID-19 acknowledged the vulnerability of women during the pandemic due to their predominance in the health care workforce, the primary caregiving roles that they play, and their disproportionate unpaid domestic work burden [34]. The results in South Africa emphasise women’s potential vulnerability and draw attention to the need for more intentional efforts to ensure that a gender-sensitive approach is taken while delivering universal health care and that a deeper, intersectional understanding of reasons for limited demand and access is required during crises. This is particularly relevant in the South African context as the move from a two-tiered system (public and private health care) to the universal National Health Insurance Fund began in 2016 and will continue over the next decade [29].

The intersection of socioeconomic circumstances with gendered access to health care found in this study is alarming, as it may also predict an inflexion point in health and health care access trajectory driven by the pandemic’s impact on socio-economic circumstances. This is particularly notable in the way preventive services have seemingly been deprioritised due to financial hardship in South Africa. The gendered nature of loss of employment income and educational opportunities due to the pandemic makes it more likely that any long-term consequences in those factors could translate to gender inequalities in health care access and potentially in health outcomes as a result. The connection between these social determinants and health care access pre-existed and is not a product of this pandemic. However, the pandemic may have accentuated them through widespread financial hardship for individuals and a likely disruption of mitigating factors (social safety nets, social capital, etc.) at the micro-level, as well as economic downturn at the macro level. The extent to which those safety nets can be re-established and to which gender-intentional economic recovery improves the financial circumstances of affected individuals could possibly determine the long-term impact of the pandemic on health and health care utilisation. While these findings are from only one country, the gendered impact of the pandemic has been detectable in other parts of the world. It is reasonable to expect that any jurisdiction with strict measures similar to those implemented initially in South Africa may potentially be experiencing similar differential impact among the most vulnerable.

Our work provided important insights into data availability during emergencies. Rapid surveys reliant on polling samples that may be self-weighted for a small section of population groups with non-random selection are a mainstay of data collection for pandemic response [15,16]. The pandemic has exposed a lack of data collection preparedness as ad hoc questions to address gender gaps were quickly developed and sometimes implemented without sufficient reliability or validity measurement. The gap is being filled with more guidance on standardised questions [35], but only after a large amount of data was already collected, with limited opportunity for improvement in follow-up rounds due to comparability concerns. This also applies to data that is not directly related to gender, but where sex-disaggregation can reveal insights. An important lesson emerging from stress-testing these data are the need for tested and validated rapid survey question inventories around health care access disruptions, with clear guidance for interviewers and due consideration for intra- and cross-country comparability. Conceptual frameworks for studying the gendered impact of global health crises should be developed and linked with data collection efforts. Larger sample sizes are crucial for intersectional analysis and having pre-prepared sampling frameworks and methodologies and the ability to rapidly mobilise resources for large surveys should be part and parcel of preparedness plans.

We note the strengths and limitations of this work. Our approach of using respondents as their own controls ensured that unmeasured characteristics which could determine demand for and access to health care were accounted for, improving our ability to attribute changes to the onset of the pandemic. However, limited sample sizes did not enable conclusively significant results to be demonstrated in some instances, despite apparently sizeable effects. Other limitations include the self-reported nature of responses, variable definitions of conditions across countries and respondents, and the likely underrepresentation of those with no or primary schooling and rural and poor households due to the exclusion of those who did not have phones from the survey, which was unavoidable given the pandemic restrictions. The sample size was also too small to explore reasons for lack of access in subgroups with intersecting social determinants of health that compound disadvantage – a common limitation with survey data [36,37].

## CONCLUSIONS

The COVID-19 pandemic is a continuing challenge as authorities around the world resume to impose or re-enact strict control measures over a year after its onset. Our findings indicate that the most vulnerable women should be prioritised for support in accessing health care as restrictions are being re-instated. For universal health care to be truly universal, post-pandemic health system strengthening efforts should pay more attention to preparedness with gender and intersectional equity lens. Further research should attempt to disentangle the underlying reasons for difficulties disadvantaged women face when trying to access health care or making decisions about priority services to utilise in the face of financial difficulty. Understanding these pathways can help to anticipate and mitigate long-term impacts and inform preparedness for future emergencies.

## Additional material


Online Supplementary Document
