# Supplementary material for: Gender and the impact of COVID-19 on demand for and access to health care: Intersectional analysis of before-and-after data from Kenya, Nigeria, and South Africa
Source: J Glob Health. 2022 Aug 13;12:05024. doi: 10.7189/jogh.12.05024 (PMC9373834; doi:10.7189/jogh.12.05024)
Supplement: Online Supplementary Document [file jogh-12-05024-s001.pdf]

Gender and the impact of COVID-19 on demand for and access to healthcare: intersectional analysis of before-and-after data from Kenya, Nigeria, and South Africa

Supplementary information

Safa Abdalla[1] MD, Elizabeth G Katz[1] PhD, Angela Hartley[1] MSc, Gary L. Darmstadt[1] MD

[1]Global Center for Gender Equality, Department of Pediatrics, Stanford University School of Medicine, Stanford, CA, USA

Correspondence: Safa Abdalla, MD, Department of Pediatrics, Stanford University School of Medicine, 1701 Page Mill Road, Stanford, CA 94305, USA, sabdalla@stanford.edu

Supplementary figure 1: Change in access to healthcare based on the PREMISE global data

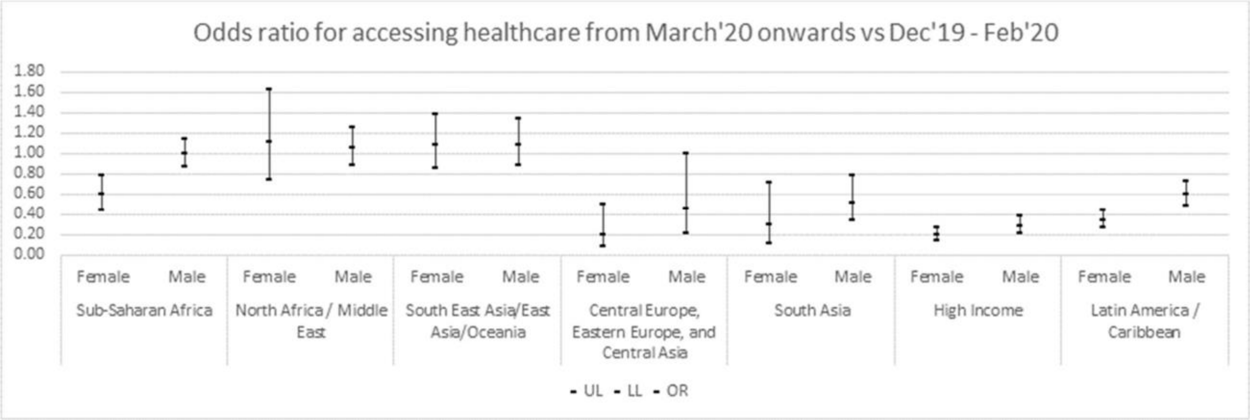


The PREMISE surveys collected data from more than 70 countries around the world, covering the experience of respondents before and after the onset of the pandemic in terms of demand and access for healthcare, including pregnancy and childbirth and child health services. The surveys were administered via mobile phone application to a non-random sample, with overrepresentation of high education groups and men. Weights were calculated for the data related to healthcare demand and access only. Country level analysis was not possible due to small sample size at country level. The regional analysis shows that the decline in healthcare access was significantly larger among women than men in Sub-Saharan Africa, and in Latin America and the Caribbean.

Supplementary table 1: Experience of financial hardship due to the pandemic by initial income and educational level in Kenya, Nigeria, and South Africa, 2020

| Characteristics | Males | Females |
|---|---|---|
| Kenya (Males weighted N=493, Females weighted N = 508) | | |
| None, primary, or secondary education | 89% | 93% |

| | | |
|---|---|---|
| Post-secondary education | 88% | 83% |
| p-value | 0.78 | 0.001 |
| Lowest tertile of income | 87% | 87% |
| Middle tertile of income | 93% | 90% |
| Highest tertile of income | 85% | 79% |
| p-value | 0.07 | 0.01 |
| Nigeria (Males weighted N=550, Females weighted N = 466) | | |
| None, primary, or secondary education | 89% | 92% |
| Post-secondary education | 93% | 86% |
| p-value | 0.09 | 0.05 |
| Lowest tertile of income | 89% | 87% |
| Middle tertile of income | 93% | 92% |
| Highest tertile of income | 92% | 84% |
| p-value | 0.51 | 0.12 |
| South Africa (Males weighted N=475, Females weighted N = 564) | | |
| None, primary, or secondary education | 70% | 69% |
| Post-secondary education | 70% | 63% |
| p-value | 0.92 | 0.21 |
| Lowest tertile of income | 66% | 63% |
| Middle tertile of income | 79% | 74% |
| Highest tertile of income | 64% | 66% |
| p-value | 0.004 | 0.04 |

*Chi-square test
